# Evaluation of laser ablation for recurrent pilonidal sinus disease: treatment success, recurrence rates, and patient outcomes

**DOI:** 10.1007/s10103-025-04525-5

**Published:** 2025-06-16

**Authors:** Ahmet Cihangir EMRAL, Sinan Efe YAZICI

**Affiliations:** 1 Atilim University, Ankara, Turkey; 2 Hacettepe University, Ankara, Turkey

**Keywords:** Pilonidal sinus disease, Laser ablation, Recurrent, Minimally invasive surgery, Postoperative outcome

## Abstract

**Purpose:**

Pilonidal sinus disease (PD) is a chronic, recurrent inflammatory condition primarily affecting the sacrococcygeal region, often resulting in discomfort, abscess formation, and recurrent disease. Various surgical interventions, including laser ablation, have been employed to treat recurrent PD. This study evaluates the efficacy of laser ablation in patients with recurrent PD, focusing on treatment success, recurrence rates, complications, and recovery outcomes.

**Methods:**

A retrospective analysis of 37 patients with recurrent pilonidal sinus disease treated with laser ablation between January 2022 and January 2025 was conducted. Preoperative data, postoperative complications, healing time, Visual Analog Scale values, and return to normal activities were collected.

**Results:**

The results showed that 70.3% of patients achieved complete healing without recurrence, while 21.6% experienced recurrence within a mean follow-up of 9.6 months. Five patients (13.5%) developed superficial infections, which were managed with local dressing. The median time for wound healing was 35 days, and patients returned to normal activities in an median of 1 day. Persistent disease was observed in 8 patients (21.6%), of whom 5 patients (62.5%) achieved full epithelialization after retreatment with laser ablation.

**Conclusion:**

The ease of application, avoidance of hospitalization, minimal postoperative care, and rapid return to daily activities make laser treatment a safe and effective therapeutic option for patients with recurrent pilonidal disease, supported by favorable outcomes and low morbidity.

## Introduction

Pilonidal sinus disease (PD) is a chronic inflammatory benign condition that commonly occurs in the sacrococcygeal region, specifically in the natal cleft. Although the etiology and pathogenesis remain unclear, the most widely accepted theory is the penetration of hair, shed from the head, neck, or back, into the natal cleft, which induces a foreign body reaction [1–3].

Although PD is more commonly observed in males, risk factors include genetics, obesity, prolonged sitting, deep natal cleft, trauma, local irritation, and excessive body hair. PD may present with symptoms or remain asymptomatic. The most common symptoms include discomfort or pain while sitting, drainage or a feeling of moisture in the natal cleft, and the development of abscesses [4, 5].

In patients with acute abscesses, the first step is drainage, whereas for chronic cases, there is no universally accepted standard treatment. Various techniques have been described for chronic PD, including excision with primary closure, various flap techniques, and non-excisional minimally invasive surgical techniques such as endoscopic pilonidal sinus treatment (EPSIT), video-assisted ablation of the pilonidal sinus (VAAPS), crystallized phenol application, and laser ablation [5–7]. Laser ablation has been shown to facilitate a quicker return to daily life, less postoperative pain, shorter hospital stays, faster recovery, and superior cosmetic results, with recurrence rates similar to those of sinus lay open and flap techniques [3, 8–10].

The aim of this study is to comprehensively evaluate the effectiveness of laser ablation treatment in patients with recurrent PD, focusing on treatment success, recurrence rates, complications, and return to normal daily life.

## Materials and methods

This retrospective study was approved by the Ethics Committee of Medicana International Hospital, Atılım University (Approval No: 21, dated 22.09.2023), in accordance with the Declaration of Helsinki. Written informed consent was obtained from all participants. Data were retrospectively analyzed from prospectively standardized clinical records. Patients who underwent laser ablation for recurrent pilonidal sinus disease (PD) between January 2022 and January 2025 were retrospectively reviewed. Postoperative follow-up was conducted weekly in the outpatient clinic until complete wound healing was achieved. After wound closure, patients were scheduled for follow-up visits at the 3^rd^, 6^th^, and 12^th^ months postoperatively. During these visits, clinical examinations were performed to evaluate wound healing and identify any complications or recurrence. Patients were advised to seek medical attention at our outpatient clinic if they experienced any abnormal changes beyond the first year. For patients with a follow-up period exceeding one year, in-person outpatient clinic evaluations were performed when possible, while others were contacted via telephone interviews.

Patients with primary diseases, a history of inflammatory bowel disease, chronic steroid use, diabetes, autoimmune disorders, prior chemotherapy or radiotherapy in the anorectal or sacrococcygeal region, malignancy, or those who had previously undergone any form of minimally invasive treatment for PD were excluded from the study. Additionally, patients with a follow-up period of less than six months were excluded to prevent statistical bias. The study included patients aged 18 years and older who met the eligibility criteria and had undergone laser ablation for recurrent PD following conventional non-minimally invasive surgical interventions.

**Fig. 1:**
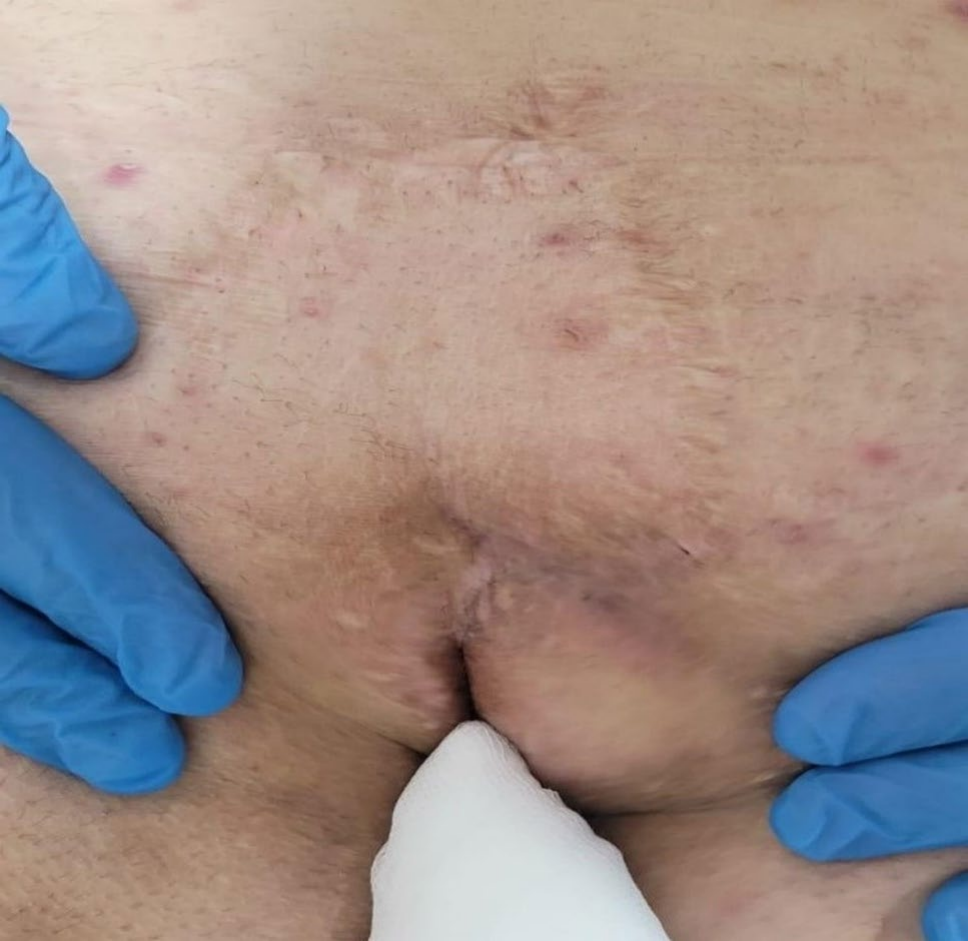
Complete epithelialization after laser ablation treatment of a patient with recurrent pilonidal sinus

Demographic data were recorded, and preoperative and perioperative information was obtained from clinical examination notes. Complete healing was defined as full epithelial closure of the sinus cavity as assessed by physical examination. Patients who failed to achieve epithelialization within two months were classified as having persistent disease. Recurrence was defined as the reappearance of an asymptomatic pit in the natal cleft or the development of an abscess or infection during the follow-up period in patients who had initially achieved complete healing. Complete epithelialization after laser ablation treatment of a patient with recurrent pilonidal sinus is shown in Fig. 1.

**Fig. 2:**
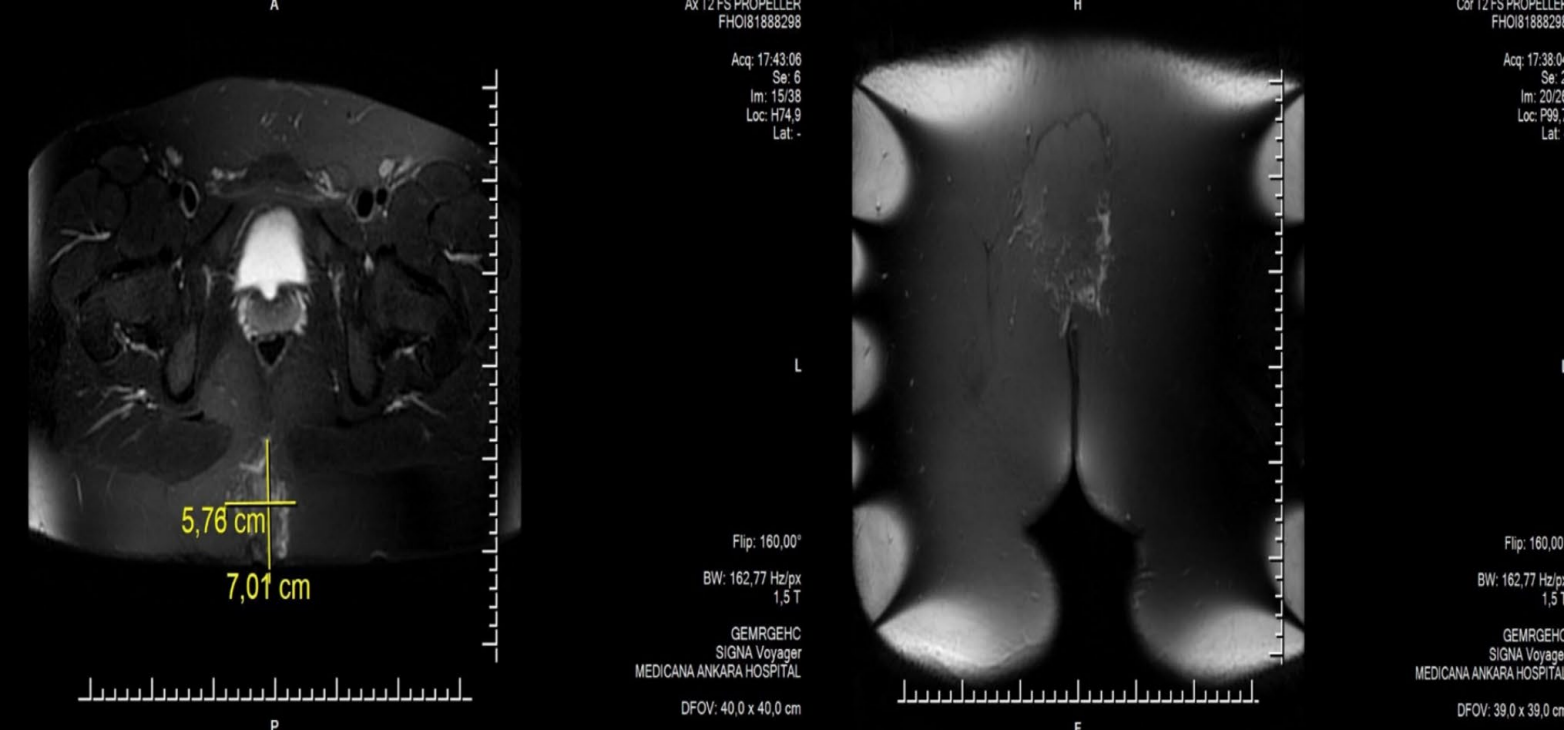
Preoperative MRI image of the patient who had previously undergone Limberg flap due to pilonidal sinus disease

**Fig. 3:**
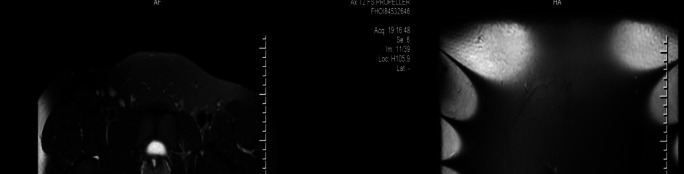
The follow-up MRI image, performed 3 months after laser therapy, showing the successful treatment outcome in a patient with recurrent pilonidal sinus previously operated using the Limberg flap technique

Preoperatively, laser depilation was recommended for all patients to address hair presence in the natal cleft, back, and neck regions if necessary. Patients presenting with an acute abscess underwent initial drainage, followed by a one-week course of antibiotic therapy (amoxicillin-clavulanic acid, 1000 mg twice daily), after which a two-week period was allowed for complete resolution of the acute infection before surgery. All patients underwent surgery in the prone position under sedation combined with local anesthesia (bupivacaine, 0.5 mg/kg). Preoperative antibiotic prophylaxis was administered as a single intravenous dose of 1 g cefazolin. In addition, all patients received a five-day course of antibiotic therapy (amoxicillin-clavulanic acid, 1000 mg twice daily) in both the preoperative and postoperative periods. Patients were discharged four hours postoperatively. In cases where the pit opening was located near the anorectal region, preoperative magnetic resonance imaging (MRI) was performed to exclude the presence of perianal fistula. Preoperative MRI image of the patient who had previously undergone Limberg flap due to pilonidal sinus disease is shown in Fig. 2. The follow-up MRI image, performed 3 months after laser therapy, showing the successful treatment outcome in a patient with recurrent pilonidal sinus previously operated using the Limberg flap technique is shown in Fig. 3.

Preoperative data, including preoperative pilonidal abscess history, treatment success, persistent disease, follow-up duration, postoperative complications, healing time (days), visual analogue scale (VAS) values on postoperative days 1^th^ and 7^th^, and time to return to normal activities (days), were analyzed. For patients with persistent disease or recurrence, a second laser ablation treatment was offered to those who consented.

### Surgical technique

#### Laser Ablation

**Fig. 4:**
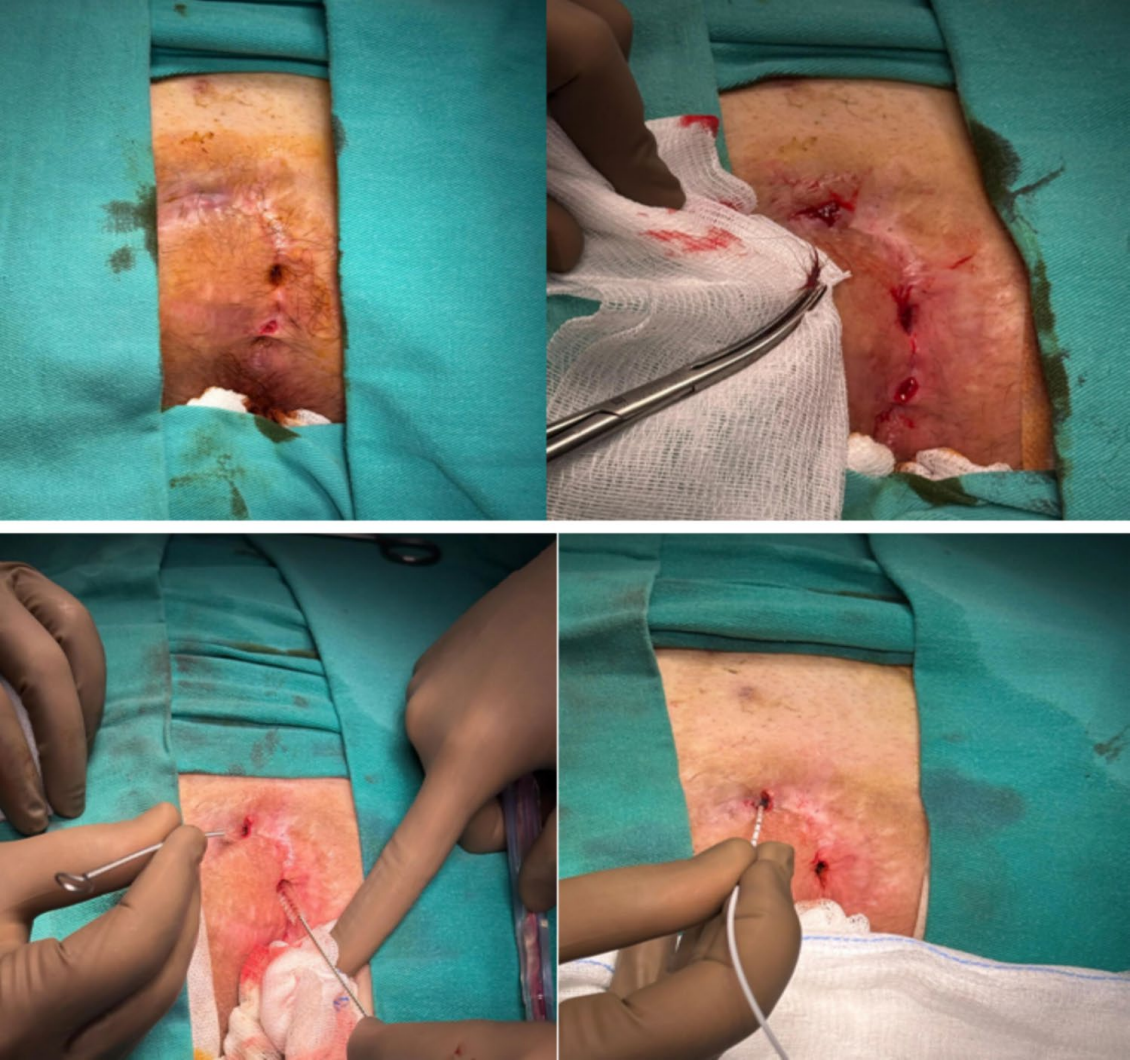
Laser ablation for the treatment of recurrent pilonidal sinus

All pit orifices were dilated using a 4 mm punch biopsy needle. The sinus cavity and pit orifices were thoroughly cleaned of hair and debris, followed by curettage. A 1470 nm diode laser probe with a 360° wavelength pattern was then introduced, and ablation was performed along each sinus tract at a continuous power setting of 12 W. The probe was withdrawn at an approximate speed of 1 mm per second. After ablation, sterile ice was applied to the pit orifice for one minute. Then standard wound dressing applied. No sutures were used. Laser ablation for the treatment of recurrent pilonidal sinus is shown in Fig. 4.

### Statistical analysis

Analyses were conducted using SPSS v27 (IBM-SPSS, Chicago, IL, USA). Distribution was assessed using skewness and kurtosis. Normally distributed data are presented as mean ± standard deviation (SD), non-normally distributed data are presented as median (min-max). Categorical variables were expressed as the number and percentage of patients. A *p*-value of less than 0.05 was considered statistically significant.

## Results

A total of 37 patients were included in the study. Laser ablation was performed as a second-line treatment in 31 patients (83.8%) and as a third-line treatment in six patients (16.2%). Among the study participants, four patients had previously undergone pit excision and primary closure followed by Limberg flap surgery due to recurrence. Additionally, one patient had undergone Karydakis flap surgery followed by Limberg flap due to recurrence, and another patient had undergone two Limberg flap surgeries due to recurrent disease. The demographic and preoperative characteristics of the patients are summarized in Table 1.

**Table 1:** Demographic and preoperative characteristics of the patients

Characteristics	Patients (*n*=37) Mean±Std. deviation (%); Median (min-max)
Age	27.75±.8
Gender	
Female	3 (8.1)
Male	34 (91.9)
BMI	29.1±2.3
Previously Abscess History	25 (67.6)
Previously Last Operation Passed Time (months)	27.3±2.6
Type of Previously Operation	
Excision with primary closure	12 (32.4)
Bascom flap	4 (10.8)
Limberg flap	12 (32.4)
Karydakis flap	9 (24.3)

A total of five patients (13.5%), including four males and one female, developed superficial surgical site infections. All of these patients were treated and healed with local dressing using 150 mg of rifampicin applied to the wound site. The female patient, aged 23 with a BMI of 28.6, had previously undergone Karydakis flap surgery but was successfully treated with laser ablation without developing persistent disease or recurrence during a nine-month follow-up. Four male patients who developed surgical site infections in the postoperative period had ages of 34, 32, 31, and 29 years, with corresponding BMIs of 30.4, 30.2, 33.9, and 29.8, respectively. The 29-year-old patient showed complete epithelialization by the 7th postoperative week without developing persistent disease, but recurrence was observed at the 6^th^ postoperative month. This patient had previously undergone pit excision with primary closure and, due to recurrence, was treated with a Limberg flap. Persistent disease developed in the other three patients, with the 34-year-old patient (BMI: 30.4) undergoing a repeat laser ablation at the 8^th^ week. Epithelialization was completed three weeks after the second procedure (11 weeks after the first procedure), and recurrence occurred at the 10^th^ postoperative month. This patient had previously undergone a Karydakis flap and, subsequently, a Limberg flap. In the other two male patients, repeat laser ablation was performed at the 10th and 11th weeks, but complete epithelialization was not achieved.

Persistent disease developed in eight patients (21.6%). One of these patients had undergone a Bascom flap, four had undergone a Limberg flap (one patient had previously undergone a Karydakis flap followed by a Limberg flap, and one patient had previously undergone pit excision with primary closure followed by a Limberg flap), and three had undergone a Karydakis flap. All patients with persistent disease were treated with repeat laser ablation, and complete epithelialization was achieved in five patients (62.5%). Three patients (8.1%) remained asymptomatic and complete epithelialization was not achieved in these cases. Of the five patients who achieved complete epithelialization after persistent disease, three experienced recurrence.

Recurrence occurred in 8 patients (21.6%). The average time to recurrence was 9.6 ± 1.7 months. In three of the patients who experienced recurrence, after being treated with laser ablation for persistent disease and achieving complete epithelialization, recurrence developed in the 11^th^, 10^th^, and 9^th^ months, respectively. Of the patients with recurrence, two had undergone a Karydakis flap, five had undergone a Limberg flap (one had previously undergone a Karydakis flap followed by a Limberg flap, and two had previously undergone pit excision with primary closure followed by a Limberg flap), and one had undergone a Bascom flap.

The postoperative outcomes of the patients are summarized in Table 2.

**Table 2:** The postoperative outcomes of patients

Characteristics	Patients (*n*=37) Mean±Std. deviation (%); Median (min-max)
Follow-up (months)	8.5±1.6
Wound Healing (days)	35 (28–77)
Complication	5 (13.5)
Postoperative day 1 VAS value	1.4±1.3
Postoperative day 7 VAS value	0 (0–6)
Return to normal activities, days	1 (1–8)
Persistent Disease	3 (8.1)
Recurrence	8 (21.6)

## Discussion

Laser treatment eliminates the epithelial tissue of the pilonidal sinus, promotes the formation of new granulation tissue, and enhances the contraction of the sinus cavity [9, 11, 12]. In their study, Pappas et al. [12] demonstrated that patients with recurrent PD healed successfully. Additionally, they found that 18 out of 23 patients (78.3%) who failed laser treatment showed healing after a second laser application. Moreover, in 27 recurrent patients treated with laser ablation, 3 (11.1%) experienced delayed healing (>60 days), with a mean healing time of 45 days (range 32–55). In this study, persistent disease was observed in 8 patients (21.6%), and after re-treatment with laser, only 3 patients (8.1%) had asymptomatic persistent disease. In persistent disease, 5 out of 8 patients (62.5%) were treated successfully with re-application of laser, which should encourage surgeons to use repeated laser treatment for patients who did not succeed with the first application. The fact that laser ablation does not require extensive tissue resection and that the laser probe can target the inflamed tract without damaging the surrounding healthy tissue may explain the low postoperative morbidity rates [9, 12].

Algazar et al. [13] in their prospective study found that while there was no superiority of Limberg flap surgery over laser ablation in terms of recurrence and complications, the laser group showed faster recovery, shorter hospital stays, shorter operation times, and lower postoperative pain compared to the Limberg group, with similar healing rates. Akyol [8] compared laser ablation and Karydakis flap patients and demonstrated, without statistically significant differences in complications or recurrence, that laser treatment was superior in terms of shorter operation times, earlier return to work, and less postoperative pain. Dessily et al. [14] reported a healing rate of 87.5%, and Horesh et al. [6] found a healing rate of 81.3% after laser ablation for PD. The recurrence rates after laser ablation in the literature range from 0–26.4% [6–9]. In their studies, Sluckin et al. [15] reported a recurrence rate of 26%, a persistence disease rate of 7%, and a success rate of 66%, with success rates increasing to 92% and 98% after second and third laser applications, respectively. In this study, 3 patients (8.1%) had persistent disease, 8 patients (21.6%) experienced recurrence, while 26 patients (70.3%) achieved complete healing without recurrence following laser treatment for recurrent PD. We believe that the lower complete healing rate in this study compared to the literature may be attributed to the fact that only recurrent patients were included, and also some of these patients (6 patients, or 16.2%) had undergone multiple surgeries previously. The literature suggests that the size of the sinus cavity, the presence of more than three pits, advanced disease and the development of postoperative infection or complications are associated with higher recurrence rates [9, 16, 17].

In recurrent PD surgery, particularly for pits located near the anal region, reoperation is the most challenging aspect of the procedure. For patients with pits near the anal region, laser treatment may present itself as an easily applicable and effective surgical option. We believe that preoperative evaluation with perianal MRI would be beneficial in these patients, especially to rule out a perianal fistula. Additionally, preoperative MRI evaluation would help map the diseased tissue in these patients. In PD disease, laser ablation treatment failure is often caused by the inability to remove foreign objects or untreated inflamed tissue inside the tract due to blind cleaning. Therefore, it is crucial to carefully clean the sinus tracts, especially to reduce the risk of recurrence [12, 18]. From this perspective, in selected cases (such as those with pits near the anal region or patients who have experienced recurrence despite multiple surgeries), preoperative radiological imaging and mapping may improve treatment success.

In conventional flap surgeries, wound dehiscence, which requires prolonged dressing and delays the patient's return to normal life, is not observed after laser treatment. While up to 25% of flap surgeries can lead to wound-related complications (such as infection, seroma, hematoma, and wound dehiscence), postoperative complication rates after laser ablation range between 0–10% [5, 7–9, 12, 16]. One of the most common complications after laser treatment is the development of a wound infection [7, 12]. As seen in this study, this complication can be managed with local wound dressings. It is believed that seroma or infection after laser treatment occurs due to the early closure of the sinus orifice opening [8, 13]. In this study, superficial infection developed in 5 patients (13.5%) and was easily managed with dressing. Although the recurrence and postoperative infection rates in this study may seem high, the fact that 67.6% of the patients had a preoperative abscess history, and that the patient group consisted largely of resistant recurrent cases, may explain the higher recurrence and postoperative complication rates observed in this study. Recurrence after laser treatment in PD usually occurs in the first postoperative year [5–9, 19]. In this study, recurrences were observed within the first year, in line with the literature.

Laser treatment in recurrent PD has proven to be effective in this study, particularly due to the absence of the need for postoperative dressing, lower postoperative pain scores, quicker return to normal life, and acceptable complication rates. Unlike flap surgery, laser ablation does not require extensive tissue excision, which results in less postoperative pain and faster recovery to normal daily activities. In this study, postoperative VAS scores and return to normal life were observed to be consistent with the literature [7–9].

Although some may believe that laser treatment may not be as meaningful in patients with previous surgical histories, particularly from a cosmetic perspective, it has still been observed that repeated flap surgeries may lead to additional poor cosmetic outcomes. Laser treatment is increasingly being recommended as the first-line approach for PD in multiple studies [6–8, 13]. Additionally, in flap techniques, subcutaneous drains may be used to manage wound infection, edema, and hematoma development [20]. The absence of the need for subcutaneous drains in laser treatment offers a significant advantage, allowing patients to return to daily life more quickly and improving patient comfort. Furthermore, for surgeons who do not frequently perform PD surgery, flap surgery may require a long learning curve [21]. In contrast, laser ablation is easy to learn and apply.

Although it is believed that not shifting the midline in laser treatment may increase the risk of recurrence in the long term, eliminating risk factors such as obesity, prolonged sitting, and excessive hair growth may be helpful in reducing recurrence. However, the major limitations of this study remain its retrospective nature, the small sample size, and the lack of long-term outcome data.

## Conclusion

Due to the ease of application, no need for hospitalization, simple postoperative care, and early return to normal life, laser treatment can be safely recommended as a treatment option for recurrent disease, even in patients with recurrence, given its successful outcomes and low, minor morbidity rates. However, further studies with longer follow-up periods and larger patient populations are necessary to confirm the long-term efficacy and durability of laser ablation as a treatment option for recurrent pilonidal sinus disease.

## Data Availability

No datasets were generated or analysed during the current study.
